# New α- and SIN γ-retrovectors for safe transduction and specific transgene expression in pancreatic β cell lines

**DOI:** 10.1186/s12896-019-0531-9

**Published:** 2019-06-17

**Authors:** Olivier Albagli, Alicia Maugein, Lukas Huijbregts, Delphine Bredel, Géraldine Carlier, Patrick Martin, Raphaël Scharfmann

**Affiliations:** 1 INSERM U1016, CNRS UMR8104, Institut Cochin, Université Paris Descartes, 123 Boulevard de Port-Royal, 75014 Paris, France; 2 Université Côte d’Azur, CNRS UMR7277 INSERM U1099, iBV (Institut de Biologie Valrose), Université Nice Sophia Antipolis, Bâtiment Sciences Naturelles; UFR Sciences, Parc Valrose, 28, avenue Valrose, 06108 Nice Cedex 2, France; 3 Present Address: Laboratoire de Recherche Translationnelle en Immunothérapie, Institut Gustave Roussy, 114 Rue Edouard Vaillant, 94800 Villejuif, France

**Keywords:** α-Retrovector, SIN γ-retrovector, Pancreatic β cell lines, EndoC-β H2 cells, Rat insulin promoter, BXV1 xenotropic retrovirus, TVA gesicles

## Abstract

**Background:**

Viral vectors are invaluable tools to transfer genes and/or regulatory sequences into differentiated cells such as pancreatic cells. To date, several kinds of viral vectors have been used to transduce different pancreatic cell types, including insulin-producing β cells. However, few studies have used vectors derived from « simple » retroviruses, such as avian α- or mouse γ-retroviruses, despite their high experimental convenience. Moreover, such vectors were never designed to specifically target transgene expression into β cells.

**Results:**

We here describe two novel α- or SIN (Self-Inactivating) γ-retrovectors containing the RIP (Rat Insulin Promoter) as internal promoter. These two retrovectors are easily produced in standard BSL2 conditions, rapidly concentrated if needed, and harbor a large multiple cloning site. For the SIN γ-retrovector, either the VSV-G (pantropic) or the retroviral ecotropic (rodent specific) envelope was used. For the α-retrovector, we used the A type envelope, as its receptor, termed TVA, is only naturally present in avian cells and can efficiently be provided to mammalian β cells through either exogenous expression upon cDNA transfer or gesicle-mediated delivery of the protein. As expected, the transgenes cloned into the two RIP-containing retrovectors displayed a strong preferential expression in β over non-β cells compared to transgenes cloned in their non-RIP (CMV- or LTR-) regulated counterparts. We further show that RIP activity of both retrovectors mirrored fluctuations affecting endogenous *INSULIN* gene expression in human β cells. Finally, both α- and SIN γ-retrovectors were extremely poorly mobilized by the BXV1 xenotropic retrovirus, a common invader of human cells grown in immunodeficient mice, and, most notably, of human β cell lines.

**Conclusion:**

Our novel α- and SIN γ-retrovectors are safe and convenient tools to stably and specifically express transgene(s) in mammalian β cells. Moreover, they both reproduce some regulatory patterns affecting *INSULIN* gene expression. Thus, they provide a helpful tool to both study the genetic control of β cell function and monitor changes in their differentiation status.

**Electronic supplementary material:**

The online version of this article (10.1186/s12896-019-0531-9) contains supplementary material, which is available to authorized users.

## Background

Transferring DNA sequences through viral vectors represents an invaluable tool to study the development and physiology of the pancreas, and explore new therapeutic strategies [1–5], and references therein]. Huge efforts have been put into targeting specific pancreatic cell types, especially among the endocrine populations clustered in islets of Langerhans [6–8]. Hence, numerous viral vectors were designed with specific, and often short, cis-regulatory DNA sequences. The goal usually is to achieve tissue-specific expression or, less frequently, to introduce a read-out for a given signaling pathway [9]. In each case, the viral vector must both achieve efficient transduction and keep the proper specificity/responsiveness of the host-derived DNA sequence.

To date, the most widely used viral vectors to transduce pancreatic cells are based on either adenoviruses, adeno-associated viruses or lentiviruses (generally HIV-1, Human Immunodeficiency Virus-1). These viral vectors provide efficient tools that allow successful and even specific gene expression in different pancreatic exocrine or endocrine sub-populations, including insulin-producing β cells [6–8, 10–13]. However, the use of vectors based on « simple » retroviruses is much rarer, and, to the best of our knowledge, was never described for specific expression in β cells. The aim of this study is thus to provide and validate new convenient and harmless viral vectors for stable transduction and specific transgene expression in β cells. We used the well known α − and γ- « simple » retroviruses as backbones and describe here two new α − and SIN (Self-Inactivating) γ-retrovectors respectively based on ASLV(A) (Avian Sarcoma and Leukosis Virus subgroup A) and MLV (Murine Leukemia Virus) retroviruses. Both harbor the RIP (Rat Insulin Promoter) as internal (transgene regulating) promoter. These retrovectors are safe, easily produced in standard BSL2 conditions and can rapidly be concentrated if needed. Importantly, in both retroviral contexts, the RIP activity is much stronger in β cells than in non-β cells and displays fluctuations that parallel those affecting the endogenous *INSULIN* gene expression.

## Results

### A SIN γ-retrovector for specific transgene expression in pancreatic β cells

#### *pPRIZ, pSERS RIP MCS and pSERS SF MCS retrovectors*

**Fig. 1:**
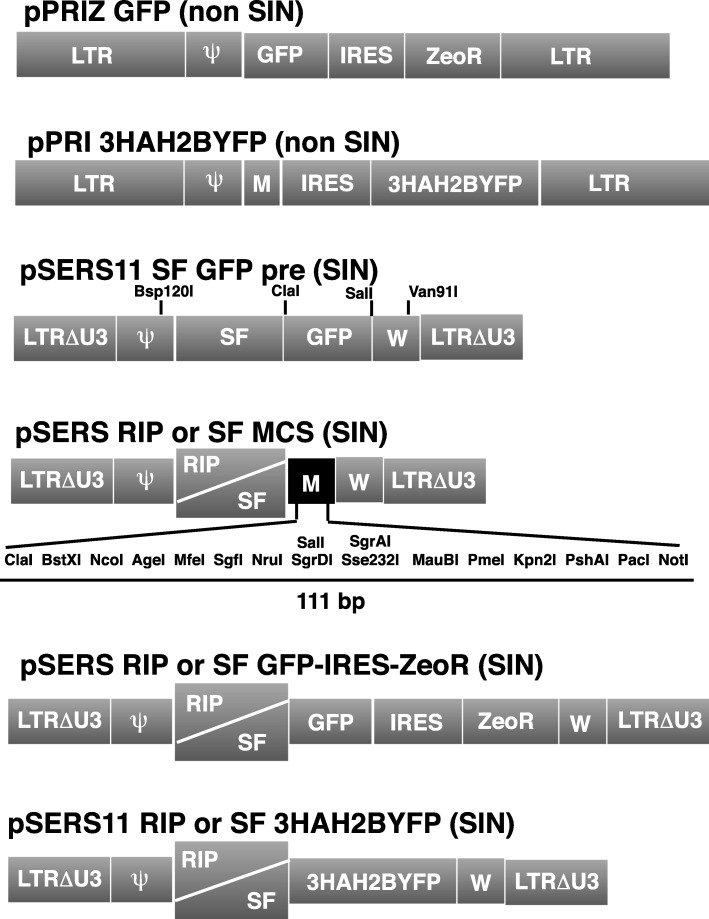
Schematic diagram of the γ-retrovectors. The integrated « proviral » form is depicted. The pSERS11 GFP pre [15] was used as a starting vector to generate a β-specific SIN γ-retrovector. LTRΔU3: Long Terminal Repeat without the U3 region (SIN deletion) Ψ: packaging sequence. RIP: 405 bp promoter of rat insulin II gene. SF: U3 enhancer/promoter region from Spleen Focus Forming Virus. M: Multi Cloning Sites are depicted in two colors (grey or black) because they are different in pPRI 3HAH2BYFP and in pSERS RIP/SF MCS (only the latter is detailed). The cDNAs subsequently inserted in these derivatives are indicated. W: Woodchuck hepatitis virus post-transcriptional regulatory element. 3HAH2BYFP: YFP fluorescent protein fused in 5′ with 3 copies of the HA epitope followed by the H2B histone. The position of the restriction sites relevant for subsequent constructs are indicated (see Methods). The different parts of the diagrams are not at scale

The pPRIZ vector has been previously described [14]. It is a non-SIN γ-retrovector harboring the Sh ble gene (thereafter referred to as ZeoR) conferring resistance to zeocin downstream of an IRES element (Fig. 1). Once retro-transcribed and integrated, the vector transcription is driven by its intact 5′ LTR, which displays a fairly ubiquitous expression. In order to design a pancreatic β-cell specific γ-retrovector, we started from the MLV-based retrovector pSERS11 SF GFP pre, a gift from Axel Schambach and Christopher Baum (Hannover Medical School, Germany) [15]. pSERS11 SF GFP pre is a SIN γ-retrovector, as its 3′ LTR (becoming 5′ LTR after retro-transcription and integration) lacks the U3 region [15]. The SF internal promoter (U3 region of SFFV LTR, Spleen Focus Forming Virus LTR), which is active in most cells, was replaced by the 405 bp RIP promoter [6, 10]. We further added several cloning sites to create the pSERS RIP MCS vector (Fig. 1). These cloning sites were also inserted into the pSERS11 SF pre GFP vector to obtain the pSERS SF MCS derivative (Fig. 1).

#### Transduction of β and non-β cell lines with the pSERS RIP SIN γ-retrovector

**Fig. 2:**
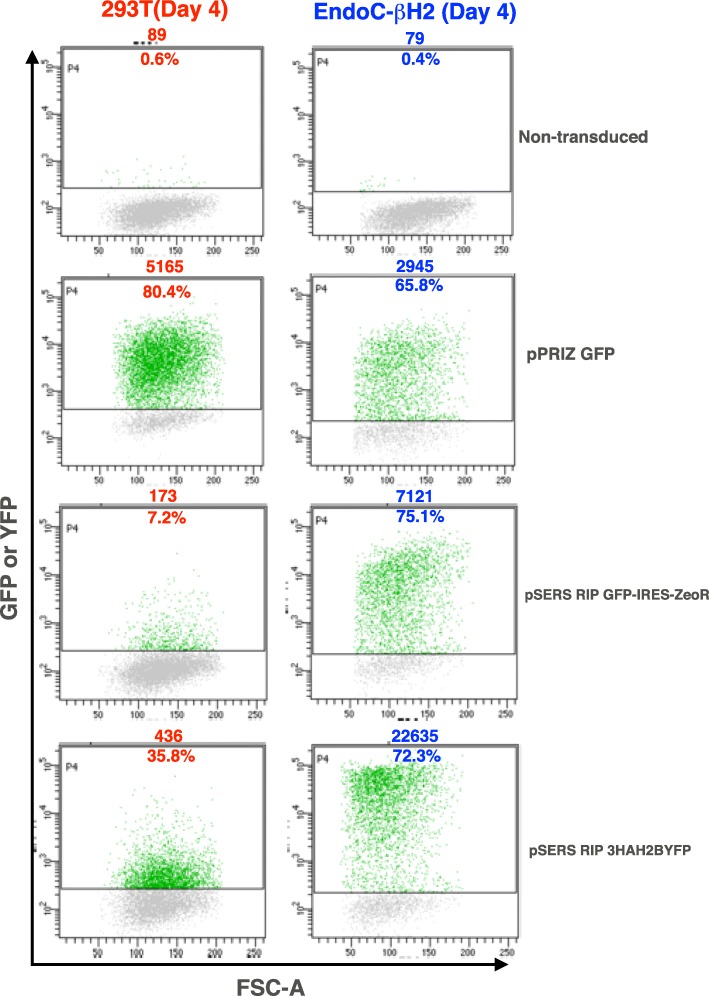
A SIN γ-retrovector mediating preferential transgene expression in β cells. Human β (EndoC-βΗ2) and non-β (293 T) cells were transduced in parallel with the indicated VSV-G pseudotyped γ-retrovectors. Cells were harvested 4 days later (without selection) and the level of GFP or YFP fluorescence was analyzed by flow cytometry. The number above each diagram indicates the mean of fluorescence of the whole population of analyzed cells. The number in the diagram is the percentage of fluorescent cells. This experiment was performed once, the two different pSERS RIP constructs giving similar results. Note that the positive threshold level is slightly higher for 293 T cells transduced with pPRIZ GFP to take into account an increase in the autofluorescence level of negative cells (lower cloud). FSC-A: Forward-Scatter-Area

Human non-β (293 T) and β cells (EndoC-βH2 [6]) were transduced with retroviral supernatants containing pSERS RIP encoding either a nuclearized and HA-tagged YFP (3HAH2BYFP) or GFP with a co-expressed selectable marker (GFP-IRES-ZeoR). pPRIZ GFP served as a control, as it also encodes a GFP-IRES-ZeoR transcript driven by the viral LTR (Fig. 1). All transductions were carried out in parallel under the same conditions, using VSV-G as envelope. After 4 days (without selection), FACS analysis showed a much stronger expression of the transgenes encoded by the RIP-containing SIN γ-retrovector in EndoC-βH2 compared to 293 T cells (Fig. 2). In contrast, transduction with pPRIZ GFP (without selection) led to a higher level of fluorescence in 293 T compared to EndoC-βΗ2 cells (Fig. 2 and Additional file 1). Stable populations of highly fluorescent β cells transduced with either pSERS RIP GFP-IRES-ZeoR or pPRIZ GFP were readily obtained upon zeocin selection (Additional file 2 and Fig. 8b). Transduction with pSERS SF GFP-IRES-ZeoR (without selection) led to a strong fluorescence level in both 293 T and EndoC-βΗ2 cells (Additional file 1). As pSERS RIP GFP-IRES-ZeoR and pSERS SF GFP-IRES-ZeoR only differ by their internal promoter, this result indicates that β cell specific expression of pSERS RIP transgene solely depends on the RIP sequence, rather than on any other feature of the construct. Finally, pSERS RIP (as well as pPRIZ and pSERS SF) also led to efficient transgene expression in transduced mouse MIN6 β insulinoma cells (Additional file 3). Thus, a SIN γ-retrovector with an internal RIP allows a stronger and preferential transgene expression in β cells compared to non-β cells (see also Additional file 10).

#### pSERS retrovectors are not mobilized by the BXV1 xenotropic retrovirus

We recently reported that EndoC-βΗ2 cells are productively infected by BXV1 (XMV-43), a mouse xenotropic endogenous γ-retrovirus [16]. As a consequence, defective γ-retrovectors are « mobilized », i.e. trans-complemented and released as xenotropic particles, raising concerns about their use in EndoC-βΗ2 cells [16]. In principle, such trans-complementation depends on the presence of the Ψ packaging signal within the γ-retrovector-encoded transcripts. Upon transient transfection, we previously verified that Ψ-deleted constructs are indeed no more trans-complemented by BXV1 [16]. However, by definition, such deleted constructs can not be used to produce viral particles.

**Fig. 3:**
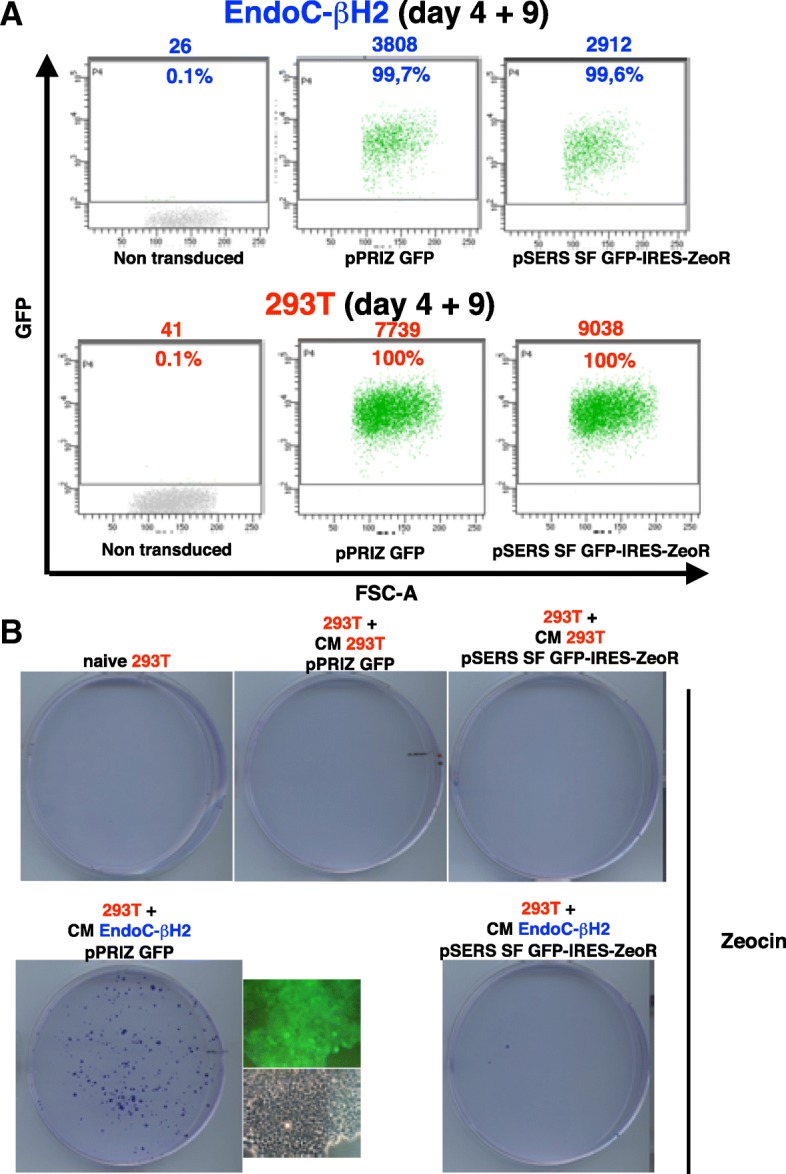
pSERS SF SIN γ-retrovector is not mobilized by the BXV1 xenotropic γ − retrovirus in EndoC-βΗ2 cells. **a**. GFP fluorescence level in transduced 293 T and EndoC-βΗ2 cells. 4 days after transduction with the indicated non-SIN (pPRIZ GFP) or SIN (pSERS SF GFP-IRES-ZeoR) γ-retrovector, 293 T and EndoC-βΗ2 cells were selected in zeocin for 9 days (day 4 + 9), then harvested and analyzed for GFP fluorescence by flow cytometry. The number above each diagram indicates the mean of fluorescence of the whole population of analyzed cells. The number in the diagram is the percentage of fluorescent cells. **b**. Conditioned medium (CM) of the transduced 293 T or EndoC-βΗ2 cells shown in A was added to « naive » 293 T cells. Transmission of zeocin resistance as a measure of γ-retrovector mobilization/trans-complementation was assessed by selecting the exposed 293 T to zeocin. GFP fluorescence was detected at various levels in most of the 293 T foci observed after their exposure to the CM of EndoC-βΗ2-pPRIZ GFP, one of them is shown. This experiment was done in duplicates, which gave similar results

The SIN (U3) deletion is aimed to dampen the transcriptional activity of the LTR [15] and hence the synthesis of Ψ-containing transcripts (Fig. 1). Therefore, most pSERS encoded mRNA should be « ignored » by the BXV1 proteins, thereby preventing trans-complementation in pSERS-transduced EndoC-βΗ2. To test this prediction, EndoC-βΗ2 cells were stably transduced with either pSERS SF GFP-IRES-ZeoR or with the non-SIN γ-retrovector, pPRIZ GFP (Fig. 1). After zeocin selection, the two EndoC-βΗ2 transduced populations showed similar GFP fluorescence levels (Fig. 3a). « Naive » 293 T cells exposed to the conditioned medium of EndoC-βΗ2 cells transduced with pPRIZ-GFP generated many zeocin-resistant (and GFP fluorescent) foci (more than 100 in two experiments) while cells exposed to the conditioned medium of EndoC-βΗ2 cells transduced with pSERS SF GFP-IRES-ZeoR gave much fewer zeocin-resistant foci (0 or 2 in two experiments) (Fig. 3b). As expected, both the supernatants of pPRIZ GFP- and pSERS SF GFP-IRES-ZeoR-transduced 293 T failed to transfer the zeocin-resistance to naive 293 T cells [16] (Fig. 3b). We also transduced EndoC-βΗ2 with both pSERS SF GFP-IRES-ZeoR and another non-SIN γ-retrovector, pPRIHy TVA, encoding hygromycin-resistance [16] and then exposed naive 293 T cells to the conditioned medium of this doubly transduced EndoC-βΗ2 population. Under these conditions, the number of hygromycin-resistant foci was well higher than that of zeocin-resistant foci (3 and 12 versus more than 500, in two experiments, respectively), confirming that non SIN γ-retrovectors are much more readily trans-complemented by BXV1 than SIN γ-retrovectors in EndoC-β Η2 cells (Additional file 4). These results indicate that the SIN γ-retrovector pSERS SF MCS, and by extension, pSERS RIP MCS, can be used in EndoC-βΗ2 cells, or in any cell contaminated with a xenotropic γ-retrovirus, without the safety concerns raised by usual, non-SIN, γ-retrovectors.

### An α-retrovector for specific transgene expression in pancreatic β cells

#### RCASBP(a) MCS, RCANBP(a) CMV MCS and RCANBP(a) RIP MCS retrovectors

RCASBP(A) and RCANBP(A) retrovectors are derived from the Rous Sarcoma Virus-A, an avian α-retrovirus belonging to ASLV(A) α-retroviridae [17]. Different pancreatic subpopulations, including β cells, have been selectively targeted with RCASBP(A) retrovectors. This was achieved through tissue-specific expression of their avian (generally quail) receptor, termed TVA, in transgenic mice [18, 19]. However, no ASLV(A)-based retrovector endowed with an intrinsic specificity for any pancreatic cell has been described to date.

**Fig. 4:**
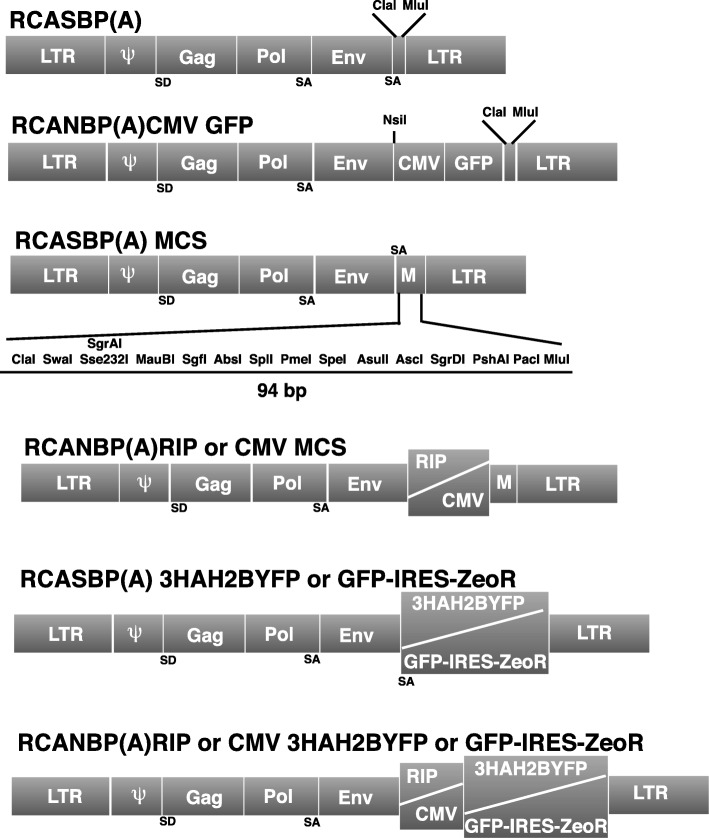
Schematic diagram of the α-retrovectors. The RCASBP(A) and RCANBP(A) CMV GFP [17–19] were used as starting vectors to generate three derivatives. All these derivatives were equipped with a large MCS (M, detailed in RCASBP(A) MCS) and one of them, RCANBP(A) RIP MCS, contains the RIP sequence as internal promoter. The cDNAs subsequently inserted in these derivatives are indicated. SD: donor splice site. SA: acceptor splice site. The different parts of the diagrams are not at scale

To design an α-retrovector for specific transgene expression in β cells, we started from RCANBP(A) CMV GFP (a gift from Stephen Hughes and Andrea Ferris, NIH/NCI, Fredericks, USA). We first removed GFP and added a new multiple cloning sites to generate RCANBP(A) CMV MCS (Fig. 4). The CMV enhancer/promoter of RCANBP(A) CMV MCS was then replaced by RIP, leading to the RCANBP(A) RIP MCS derivative (Fig. 4). Finally, the same MCS was added to the original RCASBP(A) retrovector (another gift from Stephen Hughes and Andrea Ferris) to design RCASBP(A) MCS (Fig. 4). Of note, all these constructs keep a complete 5′ LTR and should thus be all regarded as non-SIN α-retrovectors.

### Transduction of β and non-β cell lines with RCANBP(a) RIP retrovector

ASLV(A)-based retrovectors are highly efficient on chicken or quail cells. In contrast, mammalian cells are resistant because they are devoid of the ASLV(A) receptor, termed TVA. Accordingly, only mammalian cells engineered to express quail or chicken TVA can be transduced by ASLV(A)-based retrovectors [17–19]. We used two methods to express the short isoform of quail TVA in mammalian cells: 1) stable expression of the tva encoding sequence through transduction with a γ-retrovector, pPRIHy TVA, followed by hygromycin selection; 2) transient transfer of TVA through TVA-gesicles, a method adapted from the previously described transfer of the ecotropic receptor (mCAT-1) into several human cells [14, 20].

**Fig. 5:**
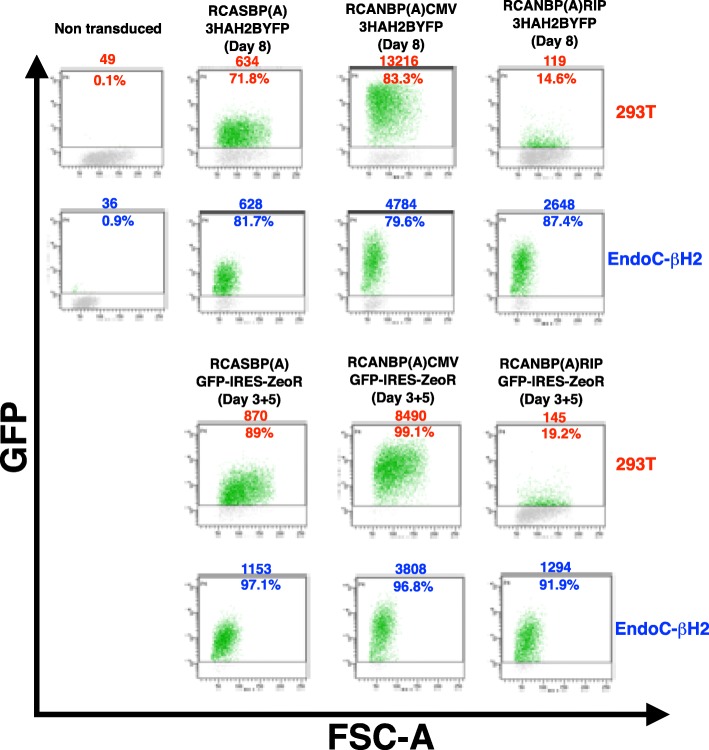
An α-retrovector mediating preferential transgene expression in β cells. EndoC-βΗ2 and 293 T cells were transduced with the indicated α-retrovectors using TVA-gesicles. Cells were harvested 8 days later and GFP fluorescence was analyzed by flow cytometry. For cells transduced with a selectable construct (GFP-IRES-ZeoR), 8 days corresponds to 3 days without zeocin and 5 days with zeocin (3 + 5). The number above each diagram indicates the mean of fluorescence of the whole population of analyzed cells. The number in the diagram is the percentage of fluorescent cells. This experiment was performed once, with two distinct reporters in the three α-retrovectors giving similar results

EndoC-βΗ2 and 293 T cells were transduced with RCANBP(A) RIP containing either 3HAH2BYFP or GFP-IRES-ZeoR using TVA-gesicles. As controls, both cell lines were also transduced with RCANBP(A) CMV MCS and RCASBP(A) MCS containing the same transgenes (Fig. 4). Both fluorescent reporters showed a much stronger expression in EndoC-βH2 cells transduced with RCANBP(A) RIP retrovector compared to 293 T cells (Fig. 5). As another comparison, while both RCANBP(A) RIP constructs were more expressed than RCASBP(A) constructs in EndoC-βH2 cells, it was the other way around in 293 T cells (Fig. 5). Moreover, while the fluorescence emitted by RCANBP(A) RIP-transduced EndoC-βΗ2 is in the same range to that of RCANBP(A) CMV-transduced EndoC-βΗ2 cells (about 1/2 to 1/3), the difference is dramatically greater in 293 T cells (about 1/50 to 1/100 fold) (Fig. 5, see also Additional files 5 and 6). Similar results were obtained in MIN6-TVA cells (Additional file 3 and Additional file 10). Thus, when delivered by an α-retrovector, RIP is much more active in β cell lines than in a non-β cell line. Of note, RIP is also barely active in chicken DF1 fibroblasts (Additional files 6 and 9), a useful feature to overcome the problem raised by the production of replicative retrovectors harboring a cytostatic or cytotoxic transgene.

Interestingly, the few fluorescent 293 T cells that were transduced with RCANBP(A) RIP 3HAH2BYFP mainly showed an evenly distributed intracellular signal, while the same construct gave the expected strictly nuclear signal in mouse or human β cells (Additional file 5). This suggests that the vector has somehow undergone « aberrant » events upon or after integration (e. g. rearrangement and/or abnormal splicing / transcriptional initiation) in most positive 293 T cells.

It may appear puzzling that despite being very dimly fluorescent, 293 T cells transduced with RCANBP(A) RIP GFP-IRES-ZeoR were resistant to zeocin (Fig. 5 and Additional file 6). Indeed, a RIP-driven bicistronic GFP-IRES-ZeoR transcript should preferentially be translated into GFP given the 5′ (Cap) position of its coding sequence [21]. This uncoupling between the expression of the two markers suggests that the resistance to zeocin does not arise from RIP-driven transcripts, but rather from the LTR-driven « genomic » transcript (5’R-U5-Gag-Pol-Env-RIP-GFP-IRES-ZeoR-U3-R3’) translated into ZeoR thanks to the IRES. This hypothesis is further supported by the fact that 293 T-TVA cells transduced with RCANBP(A) RIP ZeoR are sensitive to zeocin while cells transduced with RCASBP(A) ZeoR or RCANBP(A) CMV ZeoR are resistant (Additional file 6). Thus, RIP-driven transcription is not sufficient to confer resistance to zeocin in 293 T cells. These data confirmed that RIP brought by an α-retrovector is barely active in non-β cells.

### α-Retrovectors are not trans-complemented by BXV1

**Fig. 6:**
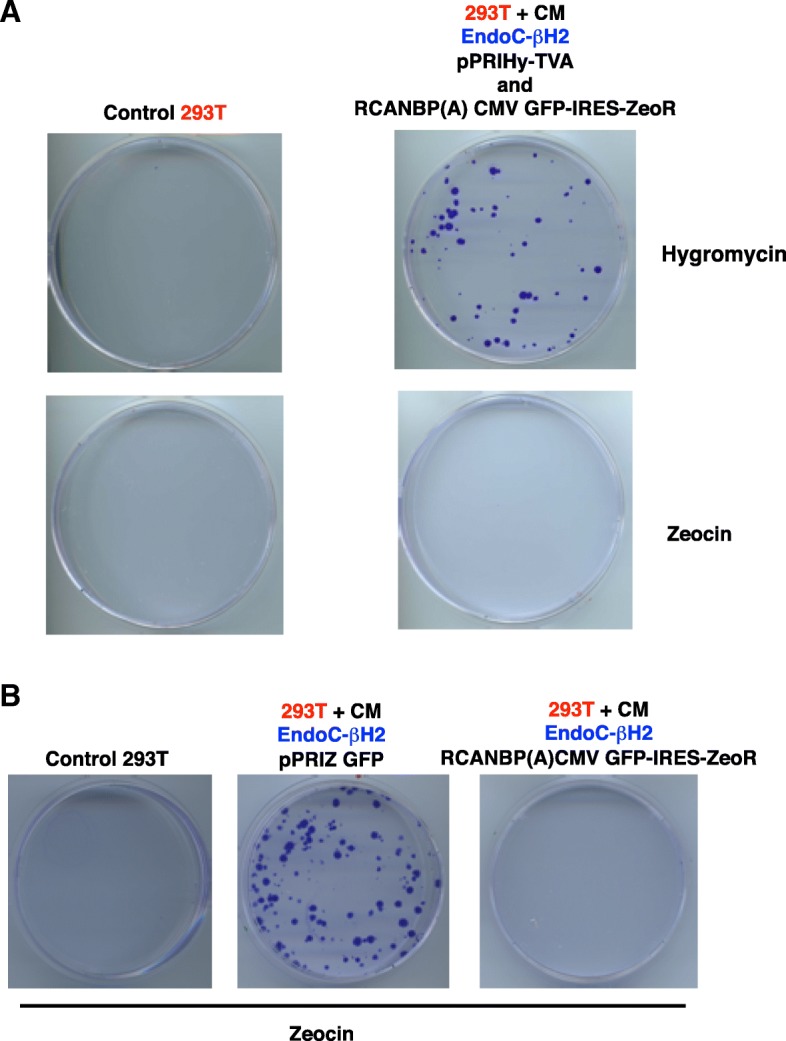
α-retrovectors are not mobilized by the BXV1 xenotropic γ − retrovirus in EndoC-βΗ2 cells. **a** EndoC-βΗ2 cells transduced with the non SIN γ-retrovector pPRIHy TVA encoding a selectable marker (HygroR) and the TVA receptor (EndoC-βΗ2-TVA) were further transduced with an α-retrovector, (RCANBP(A) CMV GFP-IRES-ZeoR) encoding a distinct selectable marker (ZeoR). Naive 293 T cells were exposed to the conditioned medium (CM) of the doubly transduced EndoC-βΗ2 cells and next divided and cultured into two petri dishes in presence of either hygromycin or zeocin. After 10 days, exposed and selected 293 T cells were fixed and colored with crystal violet. The same doubly transduced EndoC-βΗ2 derivative (EndoC-βΗ2-TVA RCANBP(A) CMV GFP-IRES-ZeoR) is used in Fig. 7**b**, showing that it is heavily positive for GFP expression. This experiment was done in duplicate, with similar results, and further confirmed using the CM of EndoC-βΗ2- transduced with both pPRIHy TVA and RCASBP(A) GFP-IRES-ZeoR) (not shown). **b** Same experiments as in (a), but naive 293 T cells were exposed to the conditioned medium (CM) of EndoC-βΗ2 harboring only one retrovector, as indicated, each encoding ZeoR. This experiment was done in duplicate, which gave similar results

**Fig. 7:**
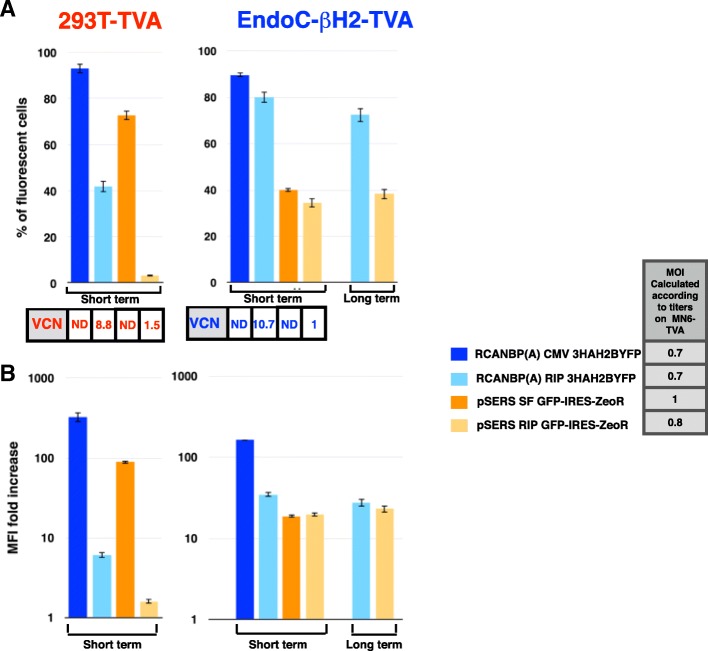
Transgene expression level and stability upon transduction with titrated RIP and non-RIP retrovectors. Two α- (RCANBP(A) CMV or RIP 3HAH2BYFP) and two γ- (pSERS SF or RIP GFP-IRES-ZeoR) retroviral supernatants, harboring either an ubiquitous (CMV or SFFV U3) or a β-specific (RIP) promoter were titrated in mouse MIN6-TVA insulinoma cells. Two human cells lines, either 293 T-TVA (left) or EndoC-βΗ2-TVA (right) cells were next transduced with the indicated retroviral supernatant, at a similar MOI (see internal legend) according to this titration. The percentage of fluorescent cells (**a**) and the MFI fold increase (**b**) were determined by flow cytometry 6 days after transduction (short term). The histogram shows the means and SD of the measures in three independent populations. The estimated mean viral copy number per cell (VCN) is indicated below each corresponding column. For clarity, VCN values have been mentioned only below the upper histogram. The same three populations of EndoC-βΗ2-TVA cells transduced with RCANBP(A) RIP 3HAH2BYFP or pSERS RIP GFP-IRES-ZeoR were cultured without selection and re-analyzed by flow cytometry for MFI fold increase and percentage of transduced cells 48 or 38 days after transduction, respectively, (long term). The MFI fold increase and percentage of fluorescent cells correspond to the mean and SD of the values measured in the three independent populations. ND: not done

ASLV(A) and MLV, although sometimes both referred to as « onco-retroviruses », are actually prototypes of two phylogenetically distant genus of the retroviridae family [22]. We thus anticipated that BXV1, a γ-retrovirus, would, at most, barely trans-complemente α-retrovectors. To test this hypothesis, we generated EndoC-βΗ2 cells sequentially transduced, first with the non-SIN γ-retrovector pPRIHy-TVA, and then with RCANBP(A) CMV GFP-IRES-ZeoR. While this derivative is doubly resistant and GFP positive (see Fig. 7b), its conditioned medium endowed naive 293 T cells with resistance to hygromycin but not to zeocin (62 and 77 versus 0 focus, respectively, in two experiments) (Fig. 6a). Concordant results were obtained when we compared the conditioned media of EndoC-βΗ2 cells transduced with either RCANBP(A) CMV GFP-IRES-ZeoR or, as a positive control, the non-SIN γ-retrovectors pPRIZ GFP (0 versus more than 100 in two experiments, respectively) (Fig. 6b). Thus, non-SIN α-retrovectors can be used in EndoC-βΗ2 cells without the safety concerns raised by non-SIN γ-retrovectors.

### Transduction at defined retroviral copy number

We next performed transductions at defined retroviral copy number of the RIP retrovectors to verify that their preferential expression in β cells is not due to higher viral titer and/or integration efficiency. pSERS RIP transgene was much more expressed in EndoC-βΗ2-TVA than in 293 T-TVA cells at a low and similar mean retroviral copy number per cell (1 vs. 1.5 respectively, Fig. 7a). Importantly, this specificity was observed according to two criterias: i) the percentage of fluorescent cells (Fig. 7a) and ii) the mean fluorescence intensity (MFI) fold increase, defined as the fluorescence intensity measured in all cells exposed to the retroviral supernatant divided by that measured in control cells that were not exposed to the retroviral supernatant (Fig. 7b). A preferential expression, albeit less pronounced, was also observed with RCANBP(A) RIP at a higher retroviral mean copy number per cell (10,7 in EndoC-βΗ2-TVA vs 8,8 in 293 T-TVA, Fig. 7a and b). Conversely, the same transgenes carried by the corresponding non-RIP retrovectors (pSERS SF and RCANBP(A) CMV) were more strongly expressed in 293 T-TVA cells than in EndoC-βΗ2-TVA cells (Fig. 7a and b). Finally, transgene expression is remarkably stable over time (38 days for pSERS RIP and 48 days for RCAN RIP) in human β cells without any selection, as demonstrated by the percentage of fluorescent cells or fluorescence intensity (Fig. 7a and b).

### RIP activity in α- and SIN γ-retroviral contexts faithfully reflects regulations affecting endogenous INSULIN gene expression

**Fig. 8:**
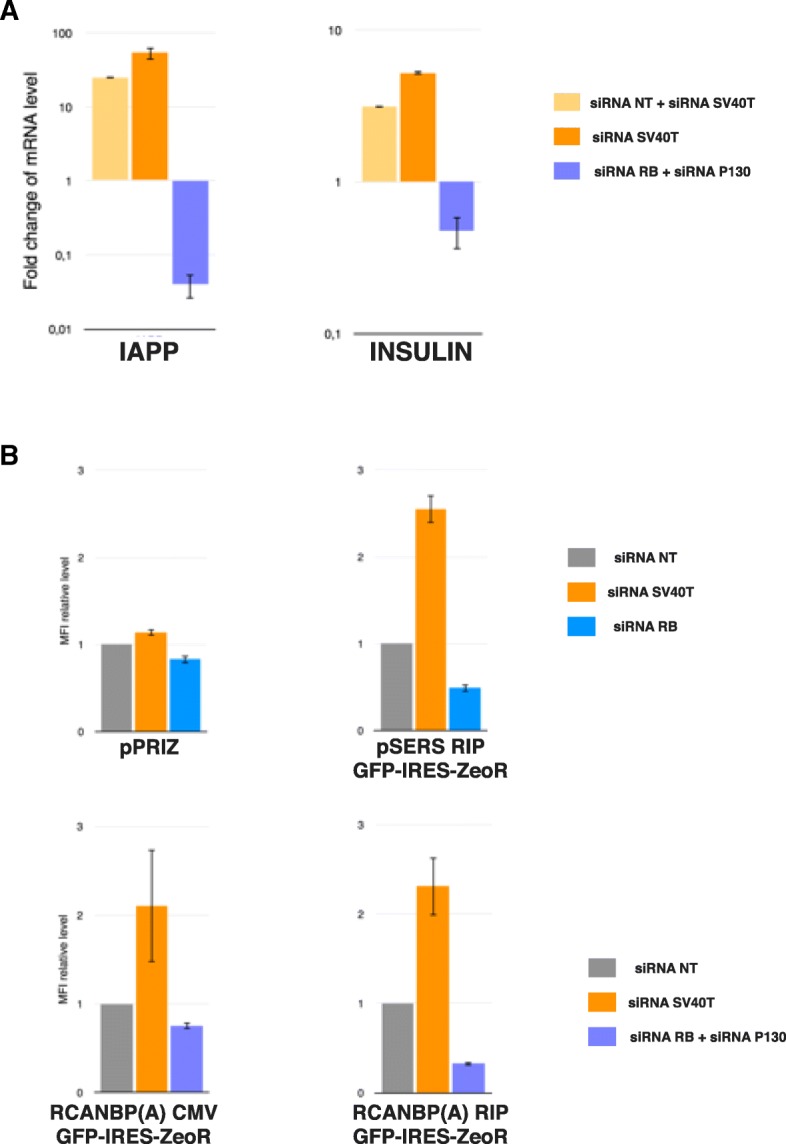
Modulations of the RIP activity in both SIN γ- and α-retrovectors tightly correlate with those affecting endogenous INSULINgene expression. a EndoC-βΗ2 cells or their derivatives stably expressing fluorescent reporters were transfected with the indicated siRNA. 6 days later, cells were harvested and changes in INSULIN and IAPP mRNA levels were determined through qRT-PCR analyses, and represented as fold change over control siRNA only (siRNA NT, Non Targeting) transfected cells. Each co-transfection (either siRNA NT + siRNA SV40T or siRNA RB + siRNA P130) was done with an equimolar mix (final concentration of 40 nM for each siRNA), while single transfection (either siRNA NT, siRNA SV40T only) was done at a final concentration of 80 nM. The results represent the mean and standard deviation (SD) of at least 3 experiments for each mRNA level analyzed, each point made in duplicate **b** EndoC-βΗ2 cells or their derivatives expressing the TVA receptor were stably transduced with the indicated γ − or α − retrovector and then transiently transfected with the indicated siRNA at a final concentration of 80 nM (80 nM for the sIRNA in single transfections, 40 nM for each siRNA in co-transfections). GFP fluorescence level was analyzed by flow cytometry 6 days after transfection. Results were expressed as fold variation in MFI (mean of fluorescence intensity) with the value in cells transfected with the siRNA arbitrarily taken as 1. The histograms show the means and SD of three (upper panels) of four (bottom panels) experiments

EndoC-βΗ2 cells have been generated through lentivector-mediated transfer of two immortalizing transgenes, hTERT and SV40 T, which can be eliminated upon sustained expression of the CRE recombinase [6]. Under these conditions, EndoC-βΗ2 cells undergo several changes akin to terminal differentiation: i) cessation of proliferation; ii) withdrawal from the cell cycle; and iii) increased expression of some key β cell genes, including IAPP (AMYLIN) and *INSULIN*, most likely at the transcriptional level [6, 14]. Most of these changes can be recapitulated upon transient knock-down of SV40 T (Fig. 8a and our unpublished data, [23]). Interestingly, knock-down of SV40 T also led to increased GFP fluorescence intensity in both pSERS RIP GFP-IRES-ZeoR and RCANBP(A) RIP GFP-IRES-ZeoR-transduced EndoC-βΗ2 cells (Fig. 7b).

The neutralization of the RB family of « pocket proteins » (RB, P107(RBL1) and P130(RBL2)) through direct physical interaction is one of the most important mechanisms underlying SV40 T induced-immortalization [24]. Quite surprisingly, the pocket proteins activity was not completely abrogated by a RIP-driven SV40 T transgene in a mouse model of insulinoma. Accordingly, Rb knock-out promotes proliferation and accelerates tumorigenesis in this experimental setting [25]. As EndoC-βΗ2 cells also harbor a RIP-driven SV40 T transgene [6], we examined whether pocket proteins depletion in these cells might cause changes reciprocal to those observed upon SV40 T depletion. Indeed, the transient knock-down of both RB and P130 mRNA (the two most highly expressed pocket proteins encoding transcripts in EndoCβΗ2 cells) reduced INS and IAPP expression (Fig. 8a). Similarly, RIP activity in the α- and SIN γ-retrovectors is reduced upon RB or RB plus P130 knock-down, respectively (Fig. 8b). Thus RIP activity again mirrored the fluctuations affecting the endogenous INS gene expression (Fig. 8b).

In summary, the variations of RIP activity in both α- and SIN γ-retroviral contexts faithfully reflected both an up-regulation and a down-regulation of INS gene expression. This tight correlation is specific to the RIP since the transfection of the same siRNA did not alter the activity of other promoters tested in similar retroviral contexts, with the exception of CMV in α-retrovector upon SV40 T depletion (Fig. 8b).

## Discussion

Our present work describes new retrovectors, termed pSERS RIP MCS and RCANBP(A) RIP MCS, designed to selectively express transgenes in pancreatic β cells. They are easy to produce and to concentrate, and harbor a large Multi Cloning Sites to facilitate DNA cloning. Both allow a strong preferential transgene expression in mammalian (mouse or human) β cells lines over non-β cell lines. Importantly, reaching a transgene expression level within the range of abundantly expressed endogenous mRNA usually is a desirable setting for practical applications. Satisfactorily, after more than 50 days without selection, transgene mRNA expression level in human β-cells transduced with either pSERS RIP or RCANBP(A) RIP was in the range of CYCLOPHILIN A (CYPA/PPIA) and INSULIN expression. Moreover, short selection of pSERS RIP GFP-IRES-ZeoR transduced cells in zeocin further increased transgene expression (Additional file 8). Our new retrovectors thus appear as convenient and promising tools to study the genetic control of β cell development or physiology. Accordingly, transgene in pSERS RIP and, to an even greater extent, in RCANBP(A) RIP, also display a strong selectivity for β cell expression upon transduction of dissociated pancreatic buds from E11-E12 mouse embryo (our unpublished data, manuscript in preparation). Although we have transferred DNA sequences encoding protein(s) in the present study, both MLV- and ASLV(A)-based retrovectors are also convenient for loss-of-function experiments [18, 19, 26–28].

Importantly, these new retrovectors are not, or extremely faintly, mobilized by the BXV1 xenotropic γ-retrovirus. Presumably, SIN γ-retrovectors are not trans-complemented because they mostly encode transcripts devoid of the Ψ packaging sequence, while α-retrovectors are too loosely related to BXV1 to use its gag- and pol-encoded proteins. Unlike non-SIN γ-retrovectors, those newly constructed retrovectors can therefore be serenely used to transduce EndoC-βΗ2 cells or any cells potentially or certainly contaminated with a xenotropic γ-retrovirus. Arguably, ASLV(A)-based retrovectors are even safer than SIN γ-retrovectors under this criteria since the formers are also very unlikely to recombine with any mammalian endogenous retroviral elements [29]. The safety of RCASBP(A)/RCANBP(A) vectors stems also from the observation that they appear defective in mammalian cells, even though they harbor the complete set of viral genes that support their efficient replication in avian cells [18, 19]. Yet, presumably because our experiments favored heavy transduction, a faint production of RCANBP(A) CMV particles was detected in transduced mammalian β or non-β cells, including cell lines, such as 293 T (Additional file 7) or rat INS1 (data not shown), not infected with xenotropic γ-retrovirus (Fig. 3b and [16]). In EndoC-βΗ2 cells, this faint production may cause the release of a few « xenotropic » RCANBP(A) CMV particles, as it bypasses the need for a true trans-complementation (i.e sharing of gag- and pol-encoded proteins) and only requires the pseudotyping of budding particles by the xenotropic Env protein. Accordingly, we sometimes observed a very limited number of zeocin-resistant foci in the experiments described in Fig. 6 (at most two) when the conditioned medium was harvested on very dense cell cultures (data not shown).

We also report here two methods to make mammalian β cells permissive to ASLV(A)-based vectors. The first method is based on transient gesicles-mediated delivery of the quail TVA receptor. We previously reported the efficiency of this method to transfer the ecotropic receptor (mCAT-1) to human β cells [14]. The second method is the stable expression of the quail tva cDNA, as previously described in transgenic mice or in other cell lines [17–19], It works also very well in human, mouse or rat β cells (Figs. 6a, 7b, Additional files 3, 5, 8, 9 and data not shown). Although more time consuming, this method allows TVA-expressing cells to stably co-express a selectable resistance gene (HygroR in our experiments). This resistance can be exploited in co-culture experiments to transduce mammalian β cells followed by a selective elimination of RCASBP(A)/RCANBP(A)-producing chicken DF1 fibroblasts (Additional file 9).

Transduction by α- and γ-retrovectors were consistently reported to be dependent on the proliferation of the target cells [18, 19]. This raises an obvious limitation for their use on mammalian β cells, which mainly are quiescent, and even probably post-mitotic in adult [30]. However, several situations throughout mammalian lifespan are associated with a significant proliferation of β cells, such as during gestation, in response to obesity (at least in rodents) and during the perinatal period (in rodents and humans) [30, 31]. β cells under such metabolic challenges should therefore be permissive to transduction with α- as well as γ-retrovectors. Experimental manipulations, such as treatment with growth hormone, could also mimic these situations and stimulate β cell proliferation [32]. Moreover, it actually is not so clear that quiescent cells are fully refractory to α-retrovirus transduction. Indeed, this assumption partly stems from observations primarily made on MLV, which requires mitosis for nuclear entry and integration of viral DNA, and was extrapolated to all « onco-retroviruses » [33]. Interestingly, early works reported that α-retrovirus (RSV) integration occurs in the absence of mitosis. In fact, either artificially arrested cell lines as well as, most importantly, naturally arrested in vitro differentiated mouse embryonic neurons, remain remarkably permissive for ASLV transduction [34]. Overall, the efficiency of α-retrovectors on non-dividing cells appears higher to that of γ-retrovectors, even though lower to that of lentivectors [33, 34]. Thus, α-retrovectors may work to some extent on differentiated (quiescent) rodent and human β cells, especially on those that have « recently » exited the cell cycle [34].

Finally, we show here that genetic manipulations that either enhance (SV40 T depletion) or decrease (RB and p130 depletion) endogenous human *INSULIN* expression, elicit similar modulations of the RIP activity in both α- and SIN γ-retroviral contexts. Transducing EndoC-βΗ2 cells with either pSERS RIP GFP-IRES-ZeoR or RCAN RIP GFP-IRES-ZeoR thus provide a convenient tool to monitor *INSULIN* gene regulation. Consequently, these derivatives can be used in high throughput screens for molecules modulating human *INSULIN* expression. A similar reporter cell line was recently designed in mouse β cells (MIN6) through the lentiviral transfer of a destabilized GFP under the control of the proximal human *INSULIN* promoter [35], indicating that its regulation can also be studied using lentivectors. However, it is worth noting that α-retroviruses integrate quite randomly throughout the genome, while lentiviruses tend to target active transcription units [36], which might not only distort normal host cell functions, but also transgene regulation. This feature of α-retroviruses is of particular interest to transfer and study short regulatory DNA regions. We thus guess that the regulation of other specific promoters of chosen pancreatic subpopulations will also be preserved upon transfer with α-retrovectors, although this remains to be tested.

Interestingly, the two means we have used to modulate *INSULIN* gene expression are unlikely to only act (directly or indirectly) on insulin (endogenous and exogenous) promoters. Indeed, the observed modulations are accompanied by variations in the expression of other β markers or in the proliferative activity of the cells. Therefore, the depletion of SV40 T or RB/P130 triggers a more global (and seemingly reciprocal) change in EndoC-βΗ2 cell phenotype of which *INSULIN* mRNA expression (or RIP activity) is only one aspect. Hence, our two RIP-GFP-transduced EndoC-βΗ2 derivatives could provide convenient read-out to follow up changes affecting not only *INSULIN* expression but also human β cell differentiation status.

## Conclusions

In the present study, we designed and validated one SIN (Self-Inactivating) γ-retrovector (MLV-based) and one α-retrovector (ASLV(A)-based), each harboring the Rat Insulin Promoter for specific transgene expression in pancreatic insulin-producing β cells. We show that: 1) these two retrovectors efficiently transduce mammalian (mouse or human) β cell lines, and we describe here a rapid method to transiently transfer the (avian) ASLV(A) receptor TVA into mammalian cells; 2) the transgenes they harbor display a strong preferential expression in β cells compared to non-β cells; 4) these two retrovectors contain a large Multi Cloning Sites, and retroviral supernatants can easily be prepared and concentrated if necessary; 5) in contrast to non-SIN γ-retrovectors, they are extremely faintly mobilized by BXV1, and can thus safely be used in human EndoC-βH β cell lines or in any cells contaminated with a mouse xenotropic γ-retrovirus; 5) the activity of the Rat Insulin Promoter in the context of both retrovectors is co-regulated with that of endogenous human *INSULIN* gene in various experimental conditions. We conclude that these new retrovectors are convenient and promising tools to study the genetic control of β cell development or physiology.

## Methods

### Constructs

#### γ-Retrovectors

We started from the SIN γ-retrovector pSERS11 SF GFP pre (a gift from Axel Schambach and Christopher Baum). Its composite 5′ promoter (RSV, Rous Sarcoma Virus U3 region fused to the SV40 virus enhancer) increases rate and processivity of transcription. Accordingly, transiently transfected 293 T cells with pSERS11 SF GFP pre produce high levels of full-length (genomic) retroviral RNA resulting in high titer supernatants [15]. We replaced part of the Ψ region, the internal SF promoter (U3 region from the SFFV retrovirus) and the GFP sequence by a synthetically synthetized DNA sequence (Eurogentec) comprising the same part of the Ψ region, the RIP 405 promoter [6, 10], and a new MCS. This linker was digested by Bsp120I and XhoI (all the restrictions enzymes were purchased from ThermoFischer or New England Biolabs) and introduced into pSERS11 SF GFP pre-digested by Bsp120I and SalI (see Fig. 1 for the location of the relevant restriction sites). pSERS SF MCS was next constructed by taking a fragment containing the MCS from pSERS RIP MCS through a ClaI and Van91I digestion (Fig. 1) which was then cloned by the same enzymes into pSERS11 SF GFP pre (which removed GFP). The GFP-IRES-ZeoR cassette was taken from RCASBP(A) GFP-IRES-ZeoR (see below) by a MreI and PacI digestion and cloned into the pSERS RIP or SF MCS digested by AgeI and PacI. pSERS RIP and SF 3HAH2BYFP were constructed by cloning a BstXI PacI fragment from pPRI 3HAH2BYFP (see below) into the vectors digested by the same enzymes. peGFP-N1 (Clontech) was digested by BamHI and NotI to harvest the GFP sequence which was inserted by the same enzymes into pPRIZ 204, a derivative of pPRIZ [14] containing a single PacI site 3′ to ZeoR. pSERS RIP and SF GFP-IRES-ZeoR were constructed by a MreI and PacI digestion of RCAN GFP-IRES-ZeoR (see below) cloned into AgeI and PacI digested vectors. pPRI 3HAH2BYFP was constructed by cloning a synthetically synthetized new 5′ part of 3HAH2BYFP containing 5′ ClaI and BstXI sites and a 3′ AgeI site (Eurogentec) into a ClaI and AgeI digested pPRIPu 3HAH2BYFP. The resulting 3HAH2BYFP sequence was digested by ClaI and DraI and inserted into RCASBP(A) MCS digested by ClaI and PshAI. Finally, 3HAH2BYFP was digested by BstXI and PacI to be cloned into pPRIG 204 (a derivative of pPRIG containing a single PacI site 3′ to GFP) which exactly replaced GFP by 3HAH2BYFP to give pPRI 3HAH2BYFP 204 (termed pPRI 3HAH2BYFP in text for simplicity). All the sequences of the final γ-retrovectors are available upon request.

#### α-Retrovectors

RCANBP(A) CMV GFP and RCASBP(A) (two gifts from Stephen Hughes and Andrea Ferris) were digested by ClaI and MluI and filled with a ClaI and MluI compatible double strand primer (Eurogentec) containing several new uniques cloning sites (Fig. 4). This step provided the RCASBP(A) MCS and RCASBP(A) CMV MCS derivatives. Next, RCANBP(A) CMV MCS was digested with NsiI and ClaI (Fig. 4) and filled with a synthetically synthetized RIP405 promoter (Eurogentec) giving rise to RCANBP(A) RIP MCS. 3HAH2BYFP was constructed by cloning a double strand DNA encoding 3HA linker (Eurogentec) compatible with ClaI in 5′ and BglII in 3′ and H2BYFP from pCMV H2BYFP (a gift from Dr. Chantal Desdouets, Institut Cochin, Paris, France) digested by BglII and MunI into ClaI and MunI digested pPRIZ 204. The resulting vector (pPRIZ 204 3HAH2BYFP) was next digested by ClaI and DraI to harvest 3HAH2BYFP, which was cloned into ClaI and PshAI digested RCASBP(A) MCS, RCANBP(A) RIP MCS and RCANBP(A) CMV MCS. RCASBP(A) GFP-IRES-ZeoR was constructed by a NarI and PacI digestion of pPRIZ 204 GFP to harvest GFP-IRES-ZeoR cloned into ClaI and PacI digested retrovector. The same cassette was inserted through a SgsI and PacI digestion into MauBI and PacI digested RCANBP(A) CMV MCS and RCANBP(A) RIP MCS. All the sequences of the final α-retrovectors are available upon request.

#### Expression vector

pCMV-TVA was constructed by taking the tva cDNA through a SmaI (a site present just 5′ to the tva cDNA in the starting vector, pENTER-TVA, a gift from Philippe Ravassard, IMC, Paris) and BglII digestion from pPRIHy-TVA [16] cloned into Eco47III and BamHI-digested peGFP-C1 (Clontech) to replace GFP by TVA.

### Production of retroviral supernatants and TVA gesicles

Ecotropic and VSV-G (Vesicular Stomatis Virus Glycoprotein)-pseudotyped γ-retrovectors were prepared as previously described [14, 16].

α-retrovectors supernatants were prepared as follows. First, 1 μg of retrovector plasmid was transfected into 30–40% confluent DF1 chicken fibroblasts (ATCC #CRL-12203) plated on six-well plates using 3 μl of X-tremeGENE 9 transfection reagent (Roche). DF1 cells were next amplified for about 8 days (following the extension of the fluorescent and/or selection marker). After this period, DF1 cells were grown to high confluency. α-retrovector supernatants were then harvested, filtered on 0.45 μm (Millipore) and concentrated (if needed) exactly like 293 T-produced γ-retrovector supernatants. Several other collects were made, if needed, during the ten following days, with an intervening 1/3 or 1/4 passage when required.

TVA gesicles were produced exactly like mCAT-1-gesicles [14] except that the pCMV-mCAT-1 expression vector was replaced by a pCMV-TVA expression vector.

All the supernatants, containing either retroviral particles or TVA-gesicles were concentrated, if necessary, on Amicon Ultra-15 (Merck) according to the recommandations of the supplier.

### Cell culture

#### Cell lines

293 T and derivatives were cultured as previously described [14]. EndoC-βΗ2 cells (and derivatives) were cultured in a Advanced-DME/F12 (Thermo Fischer) -based medium supplemented with BSA fraction V (Roche) 9,8 g/500 ml; 2 β-mercapto-ethanol (Sigma) 50 μM; sodium selenite (Sigma) 6,7 ng/ml; transferrin (Sigma) 1,2 μg/ml; nicotinamide 10 mM and antibiotics (Thermo Fischer).

The EndoC-βΗ2-TVA, 293 T-TVA, MIN6-TVA and INS1-TVA derivatives were previously described as the non-SIN γ-retrovector pPRIHy TVA encoding TVA and a selectable marker (HygroR) was used to assess trans-complementation by BXV1 in all these cell lines [16]. All of them were continuously grown in presence of hygromycin. MIN6-TVA cells were cultured in a RPMI 1640-based medium (Thermo Fischer) supplemented with glutamax (Thermo Fischer); Fetal Calf Serum (Eurobio, 10%), 2-β-mercapto-ethanol (50 microM ), sodium pyruvate (Thermo Fischer, 10 mM) and antibiotics (Thermo Fischer). Chicken DF1 fibroblasts were cultured in a F12/DMEM + Glutamine and Hepes based medium (Thermo Fischer) supplemented with fetal calf serum (Eurobio, 10%) chicken serum (Biowest 2%), sodium pyruvate (Thermo Fischer, 10 mM) and antibiotics. For selection and maintenance of selected cells, zeocin (Thermo Fischer) and hygromycin (Calbiochem) were used at 50–100 microg/ml for human β cells and at 100–200 microg/ml for rodent β cells, DF1 and 293 T cells.

#### Transduction

To compare the efficiency of γ-retrovectors on β and non-β cells, EndoC-βΗ2 or 293 T cells were plated at 10^6^ cells in 25 cm^2^ flasks. The day after, cells were exposed to 6 ml of γ-retroviral supernatants (pseudotyped by the VSV-G enveloppe) in presence of polybrene (5 microg/ml) for 4–6 h, and then grown with or without zeocin for the time lengths indicated in the main text and figures.

To compare the efficiency of the different γ-retrovectors on MIN6-TVA cells, 10^5^ cells were plated on six well plates and exposed the day after to 10 μl of concentrated retroviral ecotropic supernatant in presence of polybrene (5 μg/ml) and then grown with or without zeocin for the time lengths indicated in the main text and figures.

To compare the efficiency of α-retrovectors on β and non-β cells, EndoC-βΗ2, 293 T or MIN6-TVA cells were plated at 3 × 10^5^ cells in six-well plates. The day after, cells were exposed to 50 μl of concentrated retroviral supernatants, along with 20 μl of concentrated TVA-gesicles and polybrene (5 μg/ml) for TVA-negative cells. Transduced cells were then grown with or without zeocin for the time length indicated in the main text and figures.

For all transductions of mammalian cells with α-retrovectors, the TVA receptor was either transiently provided through gesicles (in « parental » EndoC-βΗ2 or 293 T cells), or stably expressed through prior transduction of the cells with the γ−retrovector pPRIHyTVA. In the latter cases, the cells are always denoted X-TVA (e.g. EndoC-βΗ2-TVA, MIN6-TVA, 293 T-TVA).

### Trans-complementation/mobilization assays

Trans-complementation, or retrovector mobilization, by BXV1 was assessed by exposing naive 293 T cells (10^5^ in six-well plates) to 1–2 ml of the tested conditioned medium (filtered on 0.45 μm, Millipore) for 4–6 h in presence of polybrene (5 μg/ml). The day after, cells were transferred in duplicate (and divided in two for cells transduced with two retrovectors) into the selective medium (containing zeocin or hygromycin) in 6 or 10 cm dishes to be and fixed and colored with crystal violet after 10–12 days.

### Knock-down experiments

EndoC-βΗ2 cells were plated at 40 × 10^3^ cells/cm^2^ in either six well-plates or 25cm^2^ flasks. The day after, they were transfected with the siRNA at the final concentration of 80 nM using Lipofectamine RNAiMax (Thermo-Fischer) according to the manufacturer’s instructions. siRNA RB, siRNA P130 (RBL2) or siRNA control (NT: Non targeting) were genome SMARTpool from Dharmacon(M-003296-03-0005, M-003299-03-0005, D-001206-13-20). siRNA SV40T (referred to as siRNA 2047 in [37]) was synthetized by Thermo-Fischer. Transfected cells were harvested for RNA and/or GFP fluorescence analysis 6 days after transfection.

### Cytometry

Cytometry analyzes to measure the intensity of GFP/YFP fluorescence (expressed as mean of fluorescence) were carried out as previously described [14].

### qRT-PCR analyses

Quantitative RT-PCR experiments were performed, normalized and plotted as previously described [14]. All the primers used were previously detailed [6].

### Titration of retroviral supernatants

Titration of four retroviral supernatants (pSERS SF or RIP GFP-IRES-ZeoR pseudotyed by VSV-G and RCANBP(A) CMV or RIP 3HAH2BYFP), was carried out on a reference cell line, mouse MIN6-TVA insulinoma, which efficiently expresses transgenes under the control of both ubiquitous (SF, CMV) and β-specific (RIP) promoters. Either fresh and non-concentrated (pSERS SF or RIP GFP-IRES-ZeoR) or concentrated and frozen (RCANBP(A) CMV or RIP 3HAH2BYFP) retroviral supernatant was titrated. Three doses of supernatants (25, 100 or 500 μl plus polybrene for pSERS SF or RIP retrovectors; 1, 3 or 10 μl for RCANBP(A) CMV or RIP retrovectors) were added to 2 × 10^5^ MIN6-TVA cells in six-well plates. The percentage of transduced (fluorescent) cells in each plate was measured by flow cytometry 3 days later, giving in each case two points of near-linearity. The titer was thus deduced from the value observed at the intermediate dose and used to transduce 2 × 10^5^ EndoC-βΗ2-TVA or 293 T-TVA cells in six-well plates at a defined MOI. To reach the indicated MOI (see Additional file 8), 400 μl of (fresh) pSERS SF or RIP GFP-IRES-ZeoR plus polybrene or 5 μl of (concentrated/frozen) RCANBP(A) CMV or RIP 3HAH2BYFP supernatant was added to the cells. The retroviral copy number was consistent with that expected from the MOI for the transduction of 293 T-TVA and EndoC-βΗ2-TVA cells with pSERS RIP but higher than expected for RCANBP(A) RIP 3HAH2BYFP (see below and Fig. 7). This indicates that the titration on MIN6-TVA cells is accurate for pSERS RIP retrovectors but underestimates the efficiency of the RCANBP(A) RIP retrovector on 293 T-TVA and EndoC-βΗ2-TVA cells. Yet, this discrepancy does not preclude the comparison of transgene expression level per retroviral copy in 293 T-TVA and EndoC-βΗ2-TVA transduced by RCANB(A) RIP 3HAH2BYFP, as this number of copy is similar in both cell lines (see below and Fig. 7).

### Determination of mean retroviral copy number per cell

Two EndoC-βΗ2-TVA or 293 T-TVA populations transduced with either pSERS RIP GFP-IRES-ZeoR or RCANBP(A) RIP 3HAH2BYFP were randomly chosen out of three populations generated for each vectors in each cell line. The genomic DNA of these four populations was extracted from using QIAamp DNA Micro (Qiagen). Determination of retroviral copy number was then carried out at Texcell (Evry, France). Genomic PCR was performed in duplicates for each population using the following GFP/YFP primers firstly validated on plasmids containing either GFP or YFP: CTTCAAGATCCGCCACAACATC (Fw) and GGGTCTTTGCTCAGGGCGGAC (Rev). For each PCR, the Ct (cycle threshold) value was converted to an absolute number of transgene copies using a standard curve drawn from serial dilutions of the control vectors. To evaluate the mean copy number per cell, this value was divided by the number of genomes estimated in two manners: i) the quantity of genomic DNA in the sample (50 ng) (TG/DNA); ii) the Ct obtained for the amplification of an endogenous gene, β2-microglobulin (Fw: AATTTCCTGAATTGCTATGT; Rev.: ACGGCAGGCATAYTCATC; TaqMan probe TTCAGCAARGACTGGTCTTTCTATC) in the same genome using prior calibration on defined quantities of a control plasmid (TG/β2-m). These two methods gave quite concordant results, assuming that the genome of 293 T and EndoC-βΗ2 as triploid and diploid, respectively [6, 38] and two copies of β2-microglobulin per cell in both cell lines: 0.7 (TG/DNA) and 1.5 (TG/β2-m) retroviral copy per cell for 293 T-TVA pSERS RIP GFP-IRES-ZeoR; 4,6 (TG/DNA) and 8.8 (TG/β2-m) for 293 T-TVA RCANBP(A) 3HAH2BYFP; 1 and 1 for EndoC-βΗ2-TVA pSERS RIP GFP-IRES-ZeoR; 8.3 and 10.7 for EndoC-βΗ2-TVA RCANBP(A) 3HAH2BYFP. Given this concordance and the partial tetra- or penta-ploidisation of 293 T genome [38], which may lead to somewhat underestimate the retroviral copy number calculated through TG/DNA in these cells, only the TG/β2-m results (as VCN: mean viral copy number per cell) were mentioned in the figure (Fig. 7) and text.

### Statistical analyses

Means and standard deviations were calculated using the NUMBERS software (Apple) and Student’s t tests were performed using XLSTAT v2014 (Addinsoft, Brooklyn, NY) in Additional file 10.

## Additional files

Additional file 1: Transgenes harbored by the pSERS SF γ-retrovector are efficiently expressed in both β and non-β cells. 293 T and EndoC-βΗ2 cells were transduced in parallel with either pSERS SF GFP-IRES-ZeoR or, as a control, pPRIZ GFP and GFP fluorescence was analyzed 4 days later by flow cytometry. The number above each diagram indicates the mean of fluorescence of the whole population of analyzed cells. The number in the diagram is the percentage of fluorescent cells. Note that the positive threshold level is slightly higher for 293 T cells transduced with pPRIZ GFP and pSERS SF GFP-IRES-ZeoR to take into account an increase in the autofluorescence level of negative cells (lower cloud). This experiment was done once. (DOCX 79 kb)

Additional file 2: Stable EndoC-βΗ2 populations transduced with either pPRIZ or pSERS RIP γ-retrovector. The two stable populations described in Fig. 1 (EndoC-βΗ2 transduced with either pPRIZ GFP or pSERS RIP GFP) were continuously cultured in presence of zeocin for 23 days (i. e.27 days after transduction: day 4 + 23) and GFP fluorescence was analyzed by flow cytometry. The two populations display a similar level of fluorescence intensity and are entirely constituted of fluorescent cells. Non-transduced cells are below the horizontal bar (see Fig. 3). The number above each diagram indicates the mean of fluorescence of the whole population of analyzed cells. The number in the diagram is the percentage of fluorescent cells. This experiment was done once. (DOCX 48 kb)

Additional file 3 Transduction of mouse MIN6 β cells with α- and γ-retrovectors. A. MIN6 -cell line derivative stably expressing the TVA receptor (MIN6-TVA) was transduced with the indicated γ − or α-retrovector and harvested at the indicated time for GFP fluorescence analysis by flow cytometry. When the retrovector contains a selectable transgene (ZeoR), transduced cells were selected for 3 days, starting 2 days after their exposure to the retroviral supernatant (day 2 + 3). The number above each diagram indicates the mean of fluorescence of the whole population of analyzed cells. The number in the diagram is the percentage of fluorescent cells. Note that the positive threshold level is slightly higher for cells transduced with pPRI 3HAH2BYFP to take into account an increase in the autofluorescence level of negative cells (lower cloud). These experiments were done once. (DOCX 79 kb)

Additional file 4: BXV1 efficiently mobilizes non-SIN, but not SIN, γ-retrovector in doubly transduced EndoC-βΗ2 cells. A. EndoC-βΗ2 cells transduced with the non-SIN γ-retrovector pPRIHy-TVA (EndoC-βΗ2-TVA) were further transduced with a SIN γ-retrovector (pSERS SF GFP-IRES-ZeoR). Each γ-retrovector harbors a distinct selectable marker (HygroR and ZeoR, respectively). Naive 293 T cells were next exposed to the conditioned medium (CM) of the doubly transduced EndoC-βΗ2 cells, then cultured in presence of either hygromycin or zeocin for 10 days, and finally fixed and colored with crystal violet. This experiment was done in duplicate, which gave similar results. B. The doubly transduced EndoC-βΗ2 cells display high GFP expression. This, together with their resistance to zeocin, confirms that they harbor pSERS SF GFP-IRES-ZeoR and express the corresponding transgenes. (DOCX 162 kb)

Additional file 5: Abnormal ubiquitous localization of 3HAH2BYFP in 293 T cells transduced with RCANBP(A) RIP 3HAH2BYFP. Some foci of fluorescent cells can be found in 293 T cells transduced with RCANBP(A) RIP 3HAH2BYFP. Cells within these foci often display an evenly distributed fluorescence, i.e. detectable in both the cytosol and the nucleus of the cells (left panel, upper image). In contrast, β cells (EndoC-βΗ2 or MIN6-TVA) transduced with the same retrovector display the expected nuclear fluorescence (left panel, middle and bottom images) while the same transgene in RCANBP(A) CMV-transduced 293 T also gives a strictly nuclear signal (right image). (DOCX 229 kb)

Additional file 6: RCANBP(A) RIP GFP-IRES-ZeoR transduced 293 T cells are resistant to zeocin thanks to non-RIP driven transcripts. A 293 T-TVA cells were transduced with the indicated α-retrovectors and cultured in presence of zeocin. The three retrovectors harbor ZeoR 3′ to an IRES sequence (GFP-IRES-ZeoR). After selection, cells were analyzed for GFP fluorescence intensity by flow cytometry. 293 T-TVA cells transduced with RCANBP(A) RIP GFP-IRES-ZeoR are extremely dimly fluorescent compared to the two other populations. MFI: Mean of Fluorescence Intensity. All the analyzed cells were taken into account to determine the MFI for each population, including the negative cells in RCANBP(A) RIP GFP-IRES-ZeoR-transduced 293 T-TVA cells. This experiment was done twice, which gave concordant results. B. The same three transduced 293 T-TVA cells populations (left image, right column) as well as three other populations transduced in parallel with the corresponding non-IRES containing retrovectors (left image, left column) were cultured in presence of zeocin for 7 days, then fixed and colored with crystal violet. While RCANBP(A) RIP GFP-IRES-ZeoR confers zeocin-resistance, RCAN RIP ZeoR does not. Similar observations were made in DF1 chicken fibroblasts producing the tested supernatants (middle image). In contrast, the same supernatants are equally efficient to confer zeocin resistance to MIN6-TVA β (right image). The repartition of the six populations for each six-well plate is the same, and indicated below the images. This experiment was done twice, which gave concordant results. (DOCX 172 kb)

Additional file 7: Non-SIN α-retrovectors are not totally defective in mammalian cells. 293 T-TVA cells were transduced with the non-SIN α-retrovector RCAN CMV GFP-IRES-ZeoR and cultured 12 days in presence of zeocin. Then (that is 14 days and three passages after transduction), the conditioned medium (CM) of the 293 T-TVA RCANBP(A) CMV GFP-IRES-ZeoR cells were added to naive 293 T-TVA cells, which were next exposed to a zeocin-containing medium. 2 weeks later, the receiving cells were fixed and colored with crystal violet. Some zeocin-resistant foci can be observed (middle image). As a control, naive 293 T cells (not TVA) were subjected in parallel to the same CM exposure and zeocin selection. With these cells, no zeocin resistant foci were observed (bottom image) indicating that the transmission of zeocin resistance by the conditioned medium is dependent on TVA expression in the receiving cells. As a negative control, the conditioned medium of 293 T-TVA transduced with RCASBP(A) 3HAH2BYFP (devoid of ZeoR) was also tested under the same conditions on 293 T-TVA cells (upper image). This experiment was done once and its results were confirmed using the conditioned medium of RCASBP(A) GFP-IRES-ZeoR-transduced 293 T cells (not shown). (DOCX 119 kb)

Additional file 8: Transgene expression level compared to that of two endogenous mRNA. Transgene expression level in populations of EndoC-βΗ2-TVA cells transduced with either RCANBP(A) RIP 3HAH2BYFP or pSERS RIP GFP-IRES-ZeoR (the same three populations as in Fig. 7) was analyzed through qRT-PCR. Transgene mRNA level is expressed as the cycle threshold (Ct). Cells transduced with pSERS RIP GFP-IRES-ZeoR were not exposed to zeocin (middle panel), except for one aliquot during 12 days before harvesting (right panel). The transgene expression level was compared to that of two endogenous mRNA, *CYCLOPHILIN A* and *INSULIN*, in the same transduced cells, also shown as Ct. These analyses were performed on long term transduced cells (> 50 days after transduction) and the data are the means and SD of results obtained on the three independent population for each retrovector, with each point of the qRT-PCR done in duplicate. (DOCX 37 kb)

Additional file 9: Co-culture experiments to transduce TVA-expressing mammalian β cells with RCANBPB(A) RIP retrovector. DF1 chicken fibroblasts infected with RCAN RIP GFP-IRES-ZeoR were co-cultured with either EndoC-β Η2-TVA or MIN6-TVA cells (day 0). These two derivatives express TVA together with HygroR. As the RIP is very barely active in DF1 cells, almost no fluorescent cells can be found at this time point. 2 days later, the co-culture were exposed to both zeocin, to remove untransduced β cells, and to hygromycin, to remove DF1 cells. The cells were photographed at the indicated times. After 8–10 days in hygromycin and with an intervening passage, almost all DF1 cells were eliminated. This experiment was done once and EndoC-βΗ2-TVA were transduced through co-culture with other RCASBP(A) or RCANBP(A) derivatives (not shown). (DOCX 91 kb)

Additional file 10: Comparison of RIP and non-RIP retrovector expression in β and non-β cells. Non-β 293 T-TVA and MIN6-TVA insulinoma cells were transduced with the indicated retrovectors, in each case a pair of corresponding α- (RCANBP(A)) or SIN γ − (pSERS) retrovectors harboring either a strong ubiquitous (either CMV or SF) or RIP as internal promoter. Cells were then cultured for 4 days without selection after transduction and fixed and analyzed for eGFP fluorescence by flow cytometry. Each transduction was done in triplicates. Upper panels: the percentages of fluorescent cells (means and SD) are shown. Bottom panel: for each pair of retrovectors, the relative efficiency corresponds to the percentage of fluorescent cells upon transduction with the RIP retrovector divided by the percentage observed in the same cell line transduced with the corresponding non-RIP retrovectors. Means, SD are given. For each pair of retrovectors, the ratio is significantly higher in β than in non-β cells according to a two-tailed Student’s t test. (DOCX 51 kb)

## Data Availability

All data generated or analyzed during this study are included in this published article and its supplementary information files.

## Abbreviations

3HAH2BYFP: Fusion protein with three HA epitopes (3HA), Histone H2B (H2B) and Yellow Fluorescent Protein (YFP)
AAV: Adeno Associated Virus
ASLV: Avian Sarcoma and Leukosis Virus
BXV1: B10 xenotropic virus 1 (also known as XMV-43, endogenous Xenotropic Mouse Virus 43)
CMV: Cyto Megalo Virus
hTERT: human Telomerase Reverse Transcriptase
IAPP: Islet Amyloid Poly Peptide
IRES: Internal Ribosome Entry Site
LTR: Long Terminal Repeat
mCAT-1: mouse Cationic Amino acid Transporter 1 (also known as Slc7a1, SoLute Carrier family 7, member 1)
MCS: Multi Cloning Site
MLV: Murine Leukemia Virus
MOI: multiplicity of infection
RCANBP(A): Replication-Competent ASLV long terminal repeat (LTR) No splice acceptor Bryan Polymerase subgroup A
RCASBP(A): Replication-Competent ASLV long terminal repeat (LTR) with a Spliceacceptor Bryan Polymerase subgroup A
RIP: Rat Insulin Promoter
RSV: Rous Sarcoma Virus
SFFV: Spleen Focus Forming Virus
SIN: Self-Inactivating
SV40: Simian Virus 40
TVA: Tumor Virus A
VSV-G: Vesicular Stomatitis Virus G Glycoprotein
