# Supplementary material for: New α- and SIN γ-retrovectors for safe transduction and specific transgene expression in pancreatic β cell lines
Source: BMC Biotechnol. 2019 Jun 17;19:35. doi: 10.1186/s12896-019-0531-9 (PMC6580483; doi:10.1186/s12896-019-0531-9)

Additional file 6

A

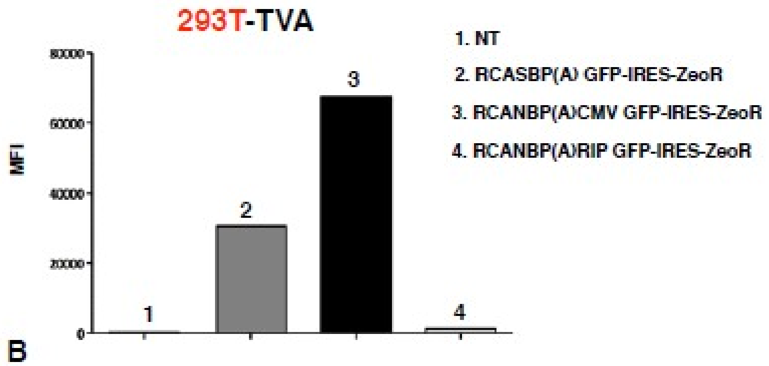

B

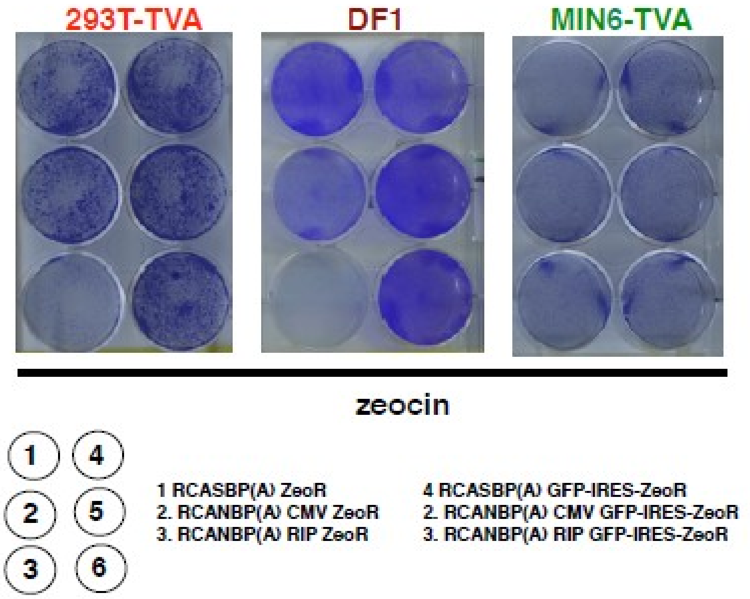

Supplement: Supplementary file 7 [file 12896_2019_531_MOESM6_ESM.docx]
