# Supplementary material for: New α- and SIN γ-retrovectors for safe transduction and specific transgene expression in pancreatic β cell lines
Source: BMC Biotechnol. 2019 Jun 17;19:35. doi: 10.1186/s12896-019-0531-9 (PMC6580483; doi:10.1186/s12896-019-0531-9)
Supplement: Supplementary file 8 [file 12896_2019_531_MOESM7_ESM.docx]

Additional file 7

293T-TVA
+ CM
293T-TVA RCASBP(A) 3HAH2BYFP

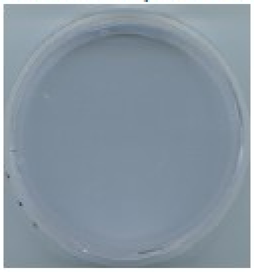

293T-TVA
+ CM
293T-TVA RCANBP(A) CMV GFP-IRES-ZeoR

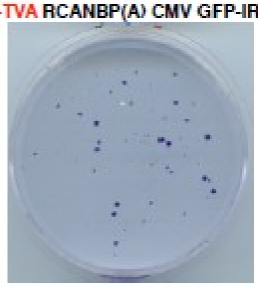

293T
+ CM
293T-TVA RCANBP(A) CMV GFP-IRES-ZeoR

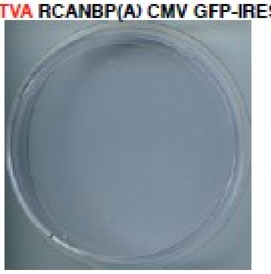

Zeocin
